# Drought stress reduces arbuscular mycorrhizal colonization of *Poncirus trifoliata* (L.) roots and plant growth promotion via lipid metabolism

**DOI:** 10.3389/fpls.2024.1452202

**Published:** 2024-09-20

**Authors:** Wei Zhang, Xilong Yin, Zengwei Feng, Xiaodi Liu, Fengwa Zhu, Honghui Zhu, Qing Yao

**Affiliations:** ^1^ Key Laboratory of Biology and Genetic Improvement of Horticultural Crops (South China), Ministry of Agriculture and Rural Affairs, Guangdong Province Key Laboratory of Microbial Signals and Disease Control, College of Horticulture, South China Agricultural University, Guangzhou, China; ^2^ Key Laboratory of Agricultural Microbiomics and Precision Application (MARA), Guangdong Provincial Key Laboratory of Microbial Culture Collection and Application, Key Laboratory of Agricultural Microbiome (MARA), State Key Laboratory of Applied Microbiology Southern China, Institute of Microbiology, Guangdong Academy of Sciences, Guangzhou, China

**Keywords:** arbuscular mycorrhiza, citrus, drought stress, lipid metabolism, *Poncirus trifoliata*, phospholipids, neutral lipids

## Abstract

Drought stress poses increasingly serious threats to agricultural production in the era of global climate change. Arbuscular mycorrhizal (AM) fungi are well-recognized biostimulants promoting plant tolerance to drought stress. Lipids are indispensable for AM fungal colonization, however, the involvement of lipid metabolism in the drought tolerance conferred by AM fungi is largely unknown. In this study, we inoculated *Poncirus trifoliata* (L.) with *Rhizophagus irregularis* DAOM197198 under no drought stress, medium drought stress and severe drought stress, with non-inoculation under respective treatments as control. Results indicated that AM fungal inoculation significantly promoted the drought tolerance of *P. trifoliata* (L.), with the effect size decreasing along with drought severity. Moreover, the effect size was significantly related to arbuscule abundance. Fatty acid profiling showed that the arbuscule abundance was determined by the AM-specific phospholipids (PLs), whose biosynthesis and delivery were inhibited by drought stress as revealed by qRT-PCR of *FatM*, *RAM1* and *STR*/*STR2*. More interestingly, AM fungal inoculation increased the lipid allocation to total PLs and the unsaturation rate of total neutral lipids (NLs), probably indicating the involvement of non-AM-specific lipids in the increased drought tolerance. Taken together, our results demonstrate that lipid metabolism in AM mediates the increased drought tolerance conferred by AM fungal inoculation, with AM-specific and non-AM-specific lipids functioning therein in different ways.

## 1 Introduction

Drought stress is one of the most important environmental constraints for agricultural production and has increasingly become severe during the past decade due to the global climate change (Arunrat et al., 2022). The use of biofertilizers as an alternative to chemical fertilizers has long been recognized as a healthier strategy for mitigating certain environmental impacts and promoting the sustainability of agricultural practices (Madawala, 2021). Substantial evidence supports the idea that beneficial microorganisms can enhance crop resilience against abiotic stressors (Kour and Yadav, 2022). Among the various candidate microorganisms, arbuscular mycorrhizal (AM) fungi have shown significant potential as biofertilizers due to their numerous advantages. AM fungi are ubiquitous soil fungi, establishing symbiotic relation with most terrestrial plants and promoting plant tolerance to drought stress (Willis et al., 2013; Wang et al., 2023a). Consequently, the study on the effects of AM fungi on plant tolerance to drought stress have greatly increased, and their positive effects have been demonstrated repeatedly (Nelsen and Safir, 1982; Ruiz-Lozano et al., 1995; Wu et al., 2008; Grümberg et al., 2015; Wang et al., 2023b).

The mechanisms underlying the enhanced drought tolerance of plants mediated by AM fungi have been extensively investigated, revealing a range of morphological, physiological, biochemical, and molecular mechanisms (Cheng et al., 2021; Wang et al., 2023a). Pioneering work by Nelsen and Safir (1982) indicated that *Glomus etunicatus* improved the drought tolerance of onion plants by enhancing phosphorus (P) nutrition; specifically, inoculated plants without P addition exhibited similar plant P status, leaf water potential, transpiration rate, and biomass as non-inoculated plants with P addition under drought conditions. Liu et al. (2024b) reported that *Claroideoglomus etunicatum* significantly increased the number and length of lateral roots, the density and length of root hairs, and the contents of abscisic acid, brassinosteroids, and gibberelins in tea plants, all of which were closely associated with the improved drought tolerance. A meta-analysis by Chandrasekaran and Paramasivan (2022) revealed that AM fungal inoculation enhances antioxidant enzymatic activity in plant tissues, playing a crucial role in alleviating drought stress, with the activities of SOD, CAT, POD, and APX increased by 32%, 23%, 28%, and 27%, respectively, resulting in a 20% reduction in H_2_O_2_ levels. Paravar and Wu (2024) found that mixed inoculation with AM fungi, which displayed the highest levels of mucilage, sugar compounds, oils, and fatty acid (FA) compositions, improved the drought resistance of plants. Furthermore, the levels of H_2_O_2_ and lipid peroxidation were significantly reduced. At the molecular level, 93% of differentially expressed genes in *Rhizophagus irregularis* were upregulated by drought, while in contrast, 78% of differentially expressed genes in plants were downregulated, including *FatM*, *RAM2*, and *STR* encoding the biosynthesis and the delivery of AM-specific lipids (Keller-Pearson et al., 2023).

AM fungi exclusively rely on plants to acquire the lipids because they cannot *de nova* biosynthesize lipids (Jiang et al., 2017). In AM fungi, phospholipids (PLs) and neutral lipids (NLs) are the key components of lipids, which occupy 25.5% and 24.2% of the total lipids in vesicles (Jabaji-Hare et al., 1984). The formation of arbuscules, the interface for nutrition exchange between plants and fungi, consume the largest proportion of PLs due to their huge surface area even in a single root cortex cell. Meanwhile, spores/vesicles contain the largest proportion of NLs probably as lipid storage for the replenishment of PLs (van Aarle and Olsson, 2003). Therefore, plant-derived lipids are essential to AM fungi for colonization and functioning. In mycorrhizal plants, *FatM and RAM2* are responsible for the biosynthesize of AM-specific lipids, while *STR/STR2* are responsible for the lipid delivery across peri-arbuscular membranes. In this scenario, the expression of these genes are highly dependent on AM fungal colonization (Keymer and Gutjahr, 2018; Keller-Pearson et al., 2023). Feng et al. (2020) demonstrated that low pH significantly limited the biosynthesis and the delivery of AM-specific lipids and accordingly reduced colonization, especially the arbuscule abundance. Zhang et al. (2024) compared the lipid consumption of three AM fungal species and found that the lipid consumption determined the effect sizes of plant growth promotion (PGP) in association with arbuscule abundance, while drought stress mediated the lipid metabolism in AM and thus the effect size. In addition, not only the AM-specific lipids but also the non-AM-specific lipids are involved in stress tolerance. For example, Wu et al. (2019) reported that the altered FA composition and saturation level by AM contributed to the enhanced drought tolerance in citrus plants. These evidence clearly indicate that the lipid metabolism in AM can play critical roles in the tolerance to diverse abiotic stresses, such as drought.

Citrus plants are the typical woody species, which are highly dependent on AM symbiosis mostly due to their scarce and short root hairs (Graham and Syvertsen, 1985). A series of studies demonstrate that AM fungal inoculation greatly enhances the drought tolerance of citrus plants (Liu et al., 2022; Wang et al., 2023b; Liu et al., 2024a). However, these studies did not link the drought tolerance to the arbuscule abundance, which is the core of AM symbiosis in term of functioning (Luginbuehl and Oldroyd, 2017). For woody plants, the roots are highly lignified and thus hard to squash the root fragments, and therefore it is difficult to clearly observe the arbuscules with the conventional method. Recently, Yin et al. (2024) developed an improved method by sectioning the lignified roots into slices of 80 μm, which enabled the assessment of arbuscule development. With this improved method, we quantified the arbuscule abundance in response to drought stress in this study, where the routinely used citrus rootstock *Poncirus trifoliata* (L.) seedlings were inoculated with AM fungus under three regimes of soil water status. We aimed to investigate (1) whether the effects of drought stress on mycorrhizal colonization, particularly on the arbuscule abundance, are related to the accumulation of AM-specific lipids in AM; and (2) whether the effects of drought stress on PGP effect size are related to the alteration in lipid profiles (AM-specific and non-AM-specific lipids) in AM.

## 2 Materials and methods

### 2.1 Experimental materials

Trifoliate orange (*Ponciru*s *trifoliata* L. Raf.) and *Rhizophagus irregularis* DAOM197198 were used to establish AM symbiosis in pot experiment. We chose *R. irregularis* DAOM197198 since this strain showed the best effect in enhancing plant drought tolerance in our previous study (Zhang et al., 2024). *R. irregularis* inocula were propagated with clover (*Trifolium repense* L.) as hosts for 4 months in the greenhouse. AM inocula consisted of fungal spores (49 spores per gram), hyphae and fragments of colonized roots. The soils from a subtropical citrus orchard (N23°9’30.3’’, E113°21’37.2’’) of South China Agricultural University were used as the experimental matrix. After air-drying at room temperature, soils were ground to pass through a 2-mm sieve. The soil chemical properties were as follows: pH 5.42, soil organic matter 37.20 g/kg, total nitrogen (N) 1.71 g/kg, total P 0.51 g/kg, total potassium (K) 10.40 g/kg, available N 111.00 g/kg, available P 15.70 g/kg, available K 108.00 g/kg.

### 2.2 Experimental design, setup and plant growth

A two-factor experimental design was applied, incorporating AM fungal inoculation (two levels: non-inoculation vs. AM fungal inoculation) and drought stress (three levels: no drought stress, medium stress, severe drought stress). Finally, there were six treatments including non-inoculation and no drought stress (N-N), non-inoculation and medium drought stress (N-M), non-inoculation and severe drought stress (N-S), AM fungal inoculation and no drought stress (A-N), AM fungal inoculation and medium drought stress (A-M), AM fungal inoculation and severe drought stress (A-S), with 5 biological replicates for each treatment. For AM fungal inoculation treatments, 100 g inocula (containing 4900 spores) were applied to each pot containing 900 soils, while sterilized inocula were used for the non-inoculation treatments with the addition of 10 mL soil filtration to maintain a homogeneous bacterial community. No drought-stressed pots were irrigated to the soil water content of 18.0%, which is equivalent to 70% of the field water holding capacity, while medium and severe drought-stressed pots were irrigated to the soil water content of 13.5% and 9.0%, respectively. We did not set a mild drought-stressed treatment (water content of 13.5%-18%) because it did not affect the plant growth of trifoliate orange seedlings in our previous experiment.

Trifoliate orange seeds were sterilized with 70% ethanol for 10 min., rinsed with clean water three times, and let germinate at 28°C in an incubator. Once the seeds germinated and the seedlings reached a height of approximately 2 cm, they were transplanted to sterilized peat moss in seedling trays. After four weeks, seedlings of approximately 5 cm in height and uniform growth were carefully selected and transplanted to pots.

All 30 pots were cultivated in the greenhouse (natural irradiance and 15-32°C temperature range). During the first 11 weeks, all plants were irrigated to 18% soil water content (namely no drought stress). From the 12^th^ week, plants were subjected to different water regimes according to the experimental design, which was achieved by weighing method. Drought stress of different levels were performed for 5 weeks, then plants were harvested.

All plants were carefully cleaned with tap water and cut into shoots and roots. Plant dry weight was recorded after oven-drying. Fine roots were used for the quantification of AM colonization, observation of arbuscules and NLs with a fluorescence microscope, lipidomic analysis, and qRT-PCR of selected genes (*FatM*, *RAM1*, *STR*/*STR2*). To compare the influence of drought stress on mycorrhizal effect, we calculated the PGP effect size as the ratio of mycorrhizal plant dry weight with inoculation to that without inoculation at each water regime.

### 2.3 Quantification of AM colonization and observation of arbuscules and NLs

Fine root fragments were sectioned with a vibrating microtome (VT1000S, Leica, Germany) according to Yin et al. (2024) and stained with trypan blue (Phillips and Hayman, 1970). AM colonization was observed under a biological microscope (Olympus BX53, Japan), and the quantification was performed with the computer program “Mycocalc” (https://www2.dijon.inra.fr/mychintec/Mycocalc-prg/download.html) according to Trouvelot et al. (1986), where the colonization frequency, total colonization intensity, relative colonization intensity, total arbuscule abundance, and relative arbuscule abundance refer to the frequency of mycorrhiza in total root fragments, intensity of mycorrhizal colonization in total root fragments, intensity of mycorrhizal colonization in colonized root fragments, arbuscule abundance in total root fragments, and arbuscule abundance in colonized root fragments, respectively.

To observe the dynamics of arbuscules and NL accumulation, double fluorescent staining of AM fungal structures and NLs was performed using WGA488 (Thermo Fisher Scientific, Shanghai, China) and Nile red (Sigma-Aldrich) (Yin et al., 2024). Root slices initially underwent an incubation in 50% ethanol for 1 hour. This was followed by a 2-hour incubation in 20% KOH at 95°C, after which the samples were washed with PBS buffer. The samples were then incubated with WGA 488 (2 mg/mL in PBS) in the dark for 30 min., with intermittent hand-shaking to ensure proper mixing. A vacuum was applied to facilitate stain penetration into the cells. Subsequently, Nile red (1 mg/mL in methanol) was added and the samples were incubated further at 50°C for an additional 30 min. Finally, after five washes with PBS buffer, the samples were mounted on slides and sealed using anti-fade mounting medium (Sangon Biotech, Shanghai, China).

### 2.4 Lipid extraction and quantification

Fatty acids were extracted from 100 mg of lyophilized roots or soils according to the method described by Feng et al. (2020). Briefly, the samples were lyophilized, ground into a fine powder, and then extracted using a mixture containing chloroform, methanol, and phosphate buffer. Following two extraction processes, the supernatants were pooled, and separation was achieved using chloroform and phosphate buffer. The solvents were subsequently evaporated in a water bath at 32°C with the aid of a nitrogen gas blower. Lipids located in the chloroform phase were then passed through a solid-phase extraction column (CNW, SBEQ-CA 1354, Shanghai, China) using chloroform, acetone, and methanol. This step facilitated the fractionation of lipids into NLs, PLs, and glycolipids. After undergoing methylation, the samples were analyzed via gas chromatography (Agilent 7890B) with hydrogen gas as the carrier. The identification of FA methyl esters was accomplished using the Sherlock Microbial Identification System (MIS 4.5, MIDI, USA), and their quantification was performed utilizing an internal standard comprising FA methyl ester 19:0 (Sigma-Aldrich, 74208). The 16:1 ω5 NLs and 16:1 ω5 PLs were separately calculated as the biomarkers of AM fungal biomass (Olsson et al., 1997).

### 2.5 RNA extraction and qRT-PCR

Total RNA was extracted from 100 mg of roots using the Omega Plant RNA Kit R6827. The concentration and quality of the extracted RNA were assessed using an ultramicroscopic spectrophotometer (ThermoFisher Scientific, NanoDrop 2000) and 1% agarose gel electrophoresis. To ensure complete removal of gDNA from RNA, purification was carried out using the Omega RNa se-Free DNa se Set E1091 kit during plant RNA extraction.

Subsequently, reverse transcription PCR was utilized to synthesize cDNA by employing the Yeasen Hifair^®^ III 1st Strand cDNA Synthesis SuperMix for qPCR (gDNA digester plus) kit, with 1 μg of RNA serving as the template. Subsequently, real-time quantitative PCR (qRT-PCR) was performed using the Yeasen Hieff^®^ qPCR SYBR Green Master Mix (No Rox) kit, with 3 replicates per sample. *Actin* gene was used as an internal standard with the primer sequence by Peng et al. (2012). The primers of *FatM*, *STR*, *STR2* were designed with the online pipeline Primer-BLAST (https://www.ncbi.nlm.nih.gov/tools/primer-blast) in this study (**Supplementary Table S1**). For each qRT-PCR reaction, a mixture of 5 μL Hieff^®^ qPCR SYBR Green Master Mix (No Rox), 0.2 μL of both forward and reverse primers, 0.5 μL of 20-fold diluted cDNA, and 4.1 μL of water was prepared. The amplification process comprised 40 cycles with pre-denaturation at 95°C for 5 min, denaturation at 95°C for 10 s, and annealing at 55°C for 30 s. Quantitative analysis was carried out using a fluorescence quantitative PCR instrument (ROCHE, LightCycler 480 II), and the relative expression of the target genes was calculated utilizing the equation 2^-ΔΔCt^ (Livak and Schmittgen, 2001).

### 2.6 Data analysis and statistics

All the data presented in this study were the average of 3-5 replicates. T-test, multiple range test (MRT), two-way analysis of variance (ANOVA) and regression analysis were performed with SPSS statistics software V.21 (SPSS Inc., Chicago, IL). To visualize the effects of AM fungal inoculation and drought stress on the lipid profiles, principal coordinate analysis (PCoA) was performed was performed with the ‘pcoa’ function of the ‘ape’ and ‘ggplot2’ packages in R software (Paradis et al., 2004; Wickham, 2011).

## 3 Results

### 3.1 Variation in PGP effect size of AM fungus under different levels of drought stress

Our data indicated that both AM fungal inoculation and drought stress significantly affected the plant growth parameters (**Supplementary Figure S1**), including morphological traits and dry weight. AM fungal inoculation significantly decreased plant height, with greater effect under severe drought than under moderate drought. This effect was also evident for other morphological parameters, e.g., stem diameter, leaf number (**Table 1**). Although drought suppressed plant growth, its effect was much greater with AM fungal inoculation than with no AM fungal inoculation, especially for plant height.

**Table 1:** The effect of AM fungal inoculation and drought stress on plant growth of *Poncirus trifoliata*.

Mycorrhizal inoculation	Drought stress	Plant height (cm)	Stem diameter (mm)	Leaf number	Shoot dry weight (g)	Root dry weight (g)	R/S ratio	Plant dry weight (g)	PGP effect size (fold change)
N	N	9.83 ± 0.47 c	1.90 ± 0.11 abc	6.00 ± 1.10 bc	0.14 ± 0.02 c	0.15 ± 0.01 c	1.13 ± 0.09 abc	0.29 ± 0.02 c	—
	M	10.28 ± 0.92 c	1.57 ± 0.07 cd	5.80 ± 0.90 bc	0.14 ± 0.02 c	0.18 ± 0.02 c	1.27 ± 0.13 ab	0.32 ± 0.03 c	—
	S	9.40 ± 0.87 c	1.40 ± 0.08 d	4.80 ± 0.80 c	0.12 ± 0.03 c	0.17 ± 0.03 c	1.51 ± 0.19 a	0.29 ± 0.06 c	—
A	N	27.28 ± 2.56 a	2.31 ± 0.13 a	18.80 ± 1.10 a	0.75 ± 0.13 a	0.34 ± 0.04 ab	0.47 ± 0.03 d	1.09 ± 0.18 a	3.80 ± 0.26 a
	M	17.93 ± 0.38 b	2.14 ± 0.02 ab	17.00 ± 1.50 a	0.52 ± 0.02 ab	0.37 ± 0.02 a	0.71 ± 0.04 cd	0.88 ± 0.03 ab	2.82 ± 0.23 ab
	S	14.17 ± 2.22 bc	1.78 ± 0.08 bcd	12.00 ± 3.60 ab	0.31 ± 0.10 bc	0.22 ± 0.03 bc	0.77 ± 0.12 bcd	0.53 ± 0.13 bc	2.06 ± 0.48 b
Two-way ANOVA (*P* values)									
AMF (A)		**0.000**	**0.000**	**0.000**	**0.000**	**0.000**	**0.000**	**0.000**	—
Drought (D)		**0.001**	**0.000**	**0.049**	**0.018**	**0.044**	**0.037**	**0.030**	**0.018**
A × D		**0.001**	0.559	0.212	**0.027**	**0.034**	0.783	**0.030**	—

N and A in mycorrhizal inoculation column indicate non-inoculation and inoculation, respectively; N, M and S in drought stress column indicate no, medium and severe drought stress, respectively. Different letters in each column indicate the significance by multiple range test (Tukey’s).

The bold values mean significant differences.

Drought did not affect plant dry weight when plants were not inoculated with AM fungus, however, it significantly suppressed both shoot dry weight and root dry weight with AM fungal inoculation. On the other hand, both shoot dry weight and root dry weight were significantly improved by AM fungal inoculation (**Table 1**). Accordingly, drought significantly increased the R/S ratio while AM fungal inoculation significantly decreased it, probably indicating the fact that drought exerted a strong stress on plants, but AM fungus alleviated this stress. It is worth noting that the PGP effect size of AM fungal inoculation varied much with drought intensity. Specifically, the PGP effect size of AM fungal inoculation was 3.80 under well-watered conditions, and decreased to 2.82 and 2.06 under moderate and severe drought conditions, respectively (**Table 1**), which suggests that drought intensity determined the PGP effect size of AM fungus (*P* = 0.018).

### 3.2 Effects of different levels of drought stress on AM fungal colonization

**Figure 1:**
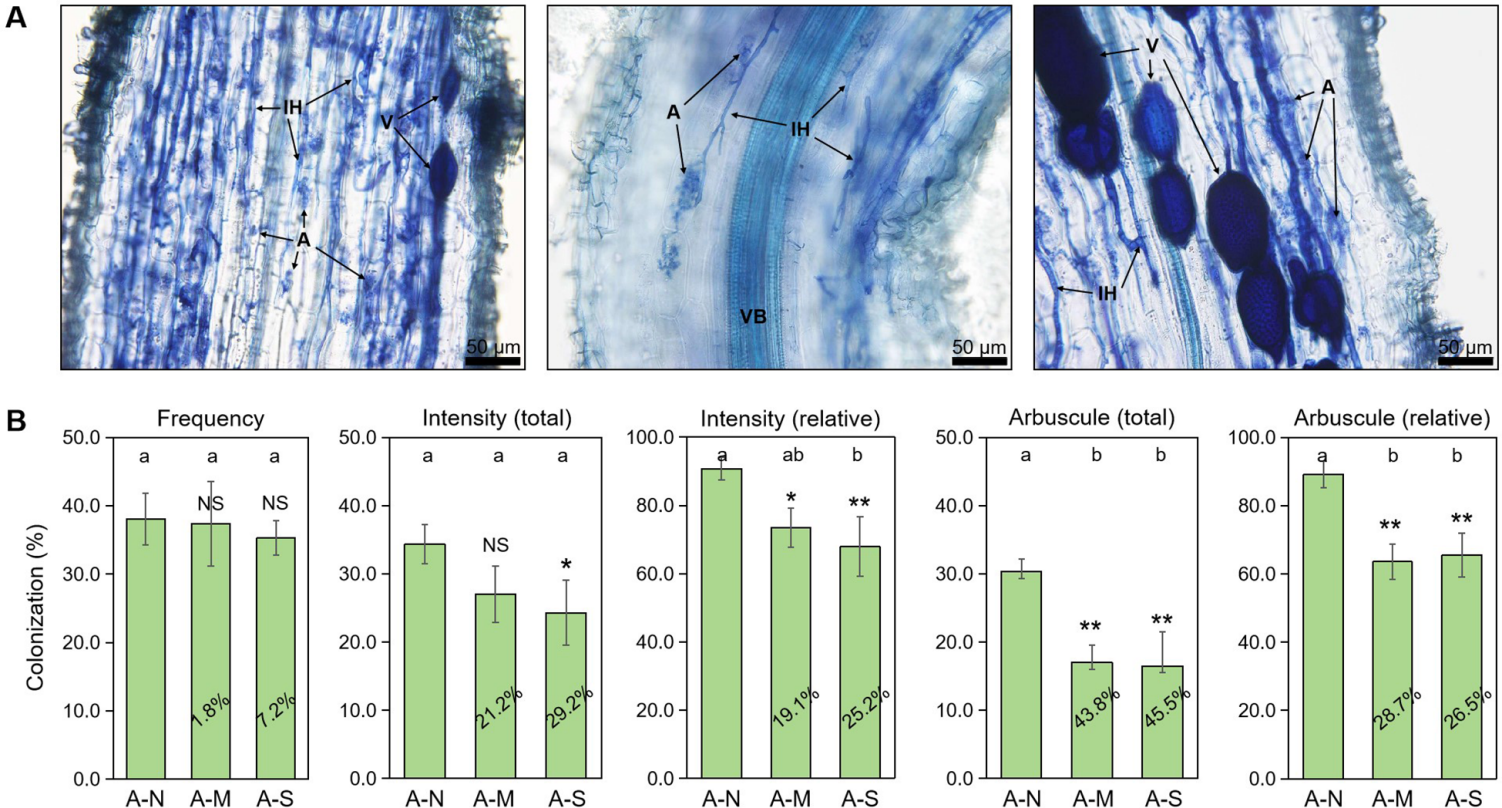
The effects of different levels of drought stress on AM fungal colonization. **(A)** Sectioned root fragments stained with trypan blue clearly showed the intraradical fungal structures in the roots of A-N treatment (200× magnification). **(B)** Quantification of the effect of drought stress, with the percentages indicating the decrease in the respective parameters. IH, internal hyphae; A, arbuscules; V, vesicles; VB, vascular bundle. A-N, A-M, and A-S indicate the treatments of no drought stress, medium drought stress, and severe drought stress with AM inoculation, respectively. Different letters in each subplot indicate the significance by multiple range test (Tukey’s). NS, and *, ** indicate no significant difference, and significant differences at *P* = 0.05, 0.01, respectively. The percentages indicate the decrease in the respective parameters of A-M or A-S compared to A-N.

Arbuscules are the site of nutrition exchange between root cells and AM fungus, and its abundance is highly indicative of the AM symbiotic effect, namely the PGP effect. We applied the sectioning method in the preparation of citrus root fragments before root staining, which has been specifically designed for the observation of arbuscules in the strongly lignified roots of woody plants (Yin et al., 2024). In the inoculated roots, arbuscules, internal hyphae and vesicles were clearly observed with trypan blue staining (**Figure 1A**). Data showed that drought stress slightly decreased the colonization frequency by <10%, and significantly decreased the colonization intensity by approximately 20%-30%. In contrast, the decrease in arbuscule abundance by drought stress was the largest, reaching 26.5%-45.5% (**Figure 1B**). This indicates that arbuscules were the most sensitive to drought stress among AM fungal structures.

### 3.3 Effects of different levels of drought stress on the lipid consumption by AM fungus

**Figure 2:**
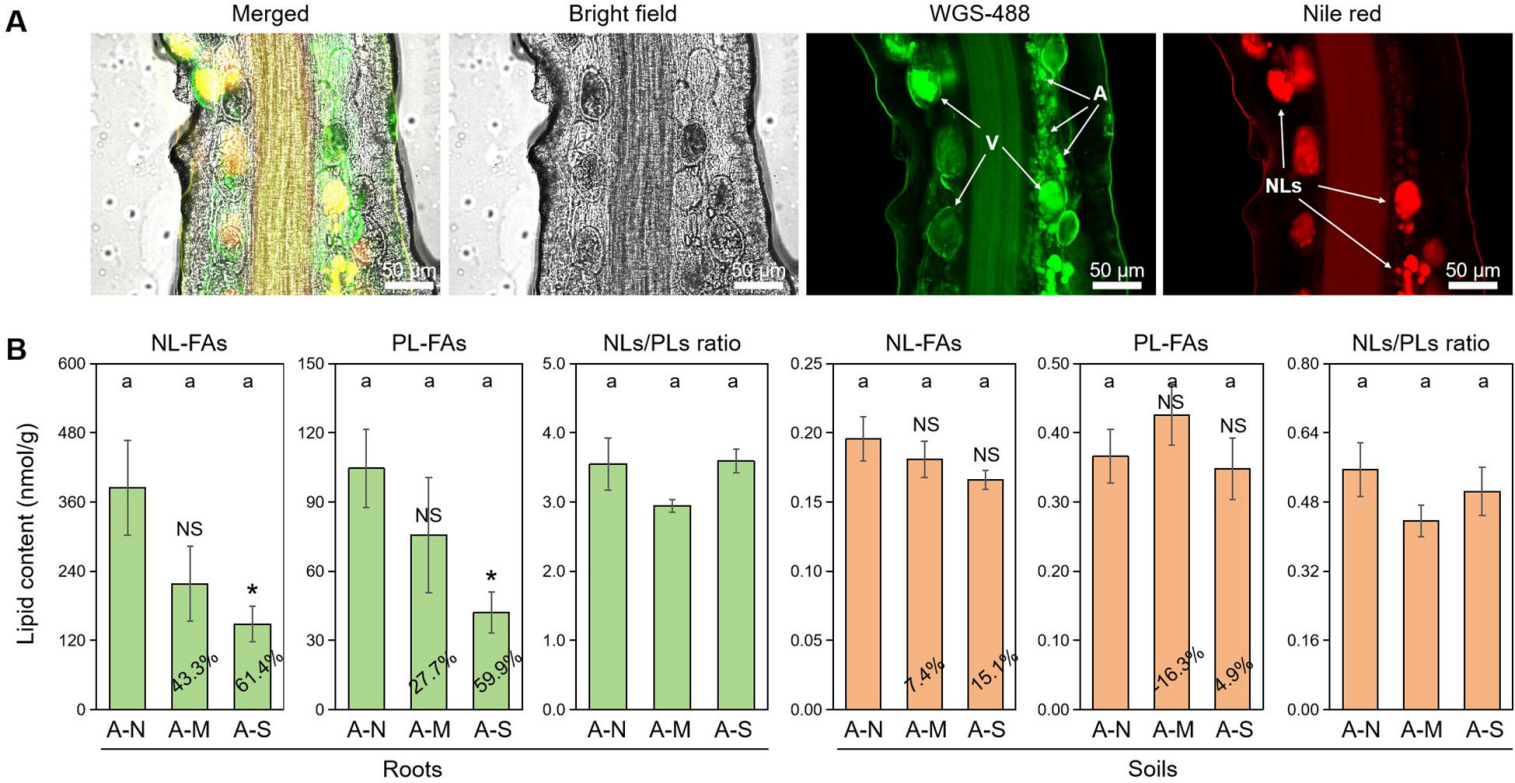
The effects of different levels of drought stress on lipid accumulation. **(A)** Sectioned root fragments stained with fluorescent probes WGA-488 and Nile red clearly showed the intraradical fungal structures and the accumulation of NLs in the roots of A-N treatment (200× magnification). **(B)** Quantification of the effect of drought stress on lipid contents in roots and soils, with the percentages indicating the decrease in the respective parameters. A, arbuscules; V, vesicles; NLs, neutral lipids. A-N, A-M, and A-S indicate the treatments of no drought stress, medium drought stress, and severe drought stress with AM inoculation, respectively. Different letters in each subplot indicate the significance by multiple range test (Tukey’s). NS and * indicate no significant difference and significant differences at *P* = 0.05, respectively. The percentages indicate the decrease in the respective parameters of A-M or A-S compared to A-N.

It is well-documented that plant-derived lipids are essential to arbuscule development, and abiotic stresses promote the degradation of arbuscules and thus the conversion of PLs to NLs (Feng et al., 2020). With the dual fluorescence staining technique, we observed the accumulation of NLs in vesicles and the development of arbuscules consuming a great deal of PLs (**Figure 2A**).

FA analysis revealed that drought stress decreased the contents of AM-specific lipids (both NL-FAs and PL-FAs) in colonized roots. Specifically, moderate drought decreased NL-FAs and PL-FAs by 43.3% and 27.7%, respectively, while severe drought decreased NL-FAs and PL-FAs by 61.4% and 59.9%, respectively, with a significant effect (**Figure 2B**). There was no difference in NLs/PLs ratio among the three treatments of drought stress, indicating that drought stress did not affect the allocation of plant-derived lipids to NLs (stored lipids) or PLs (membrane lipids). In contrast, drought stress slightly affected the contents of AM-specific lipids and NLs/PLs ration in soils, which probably suggests that drought stress does not exert influence on the lipid metabolism in the external structures of AM fungus.

**Figure 3:**
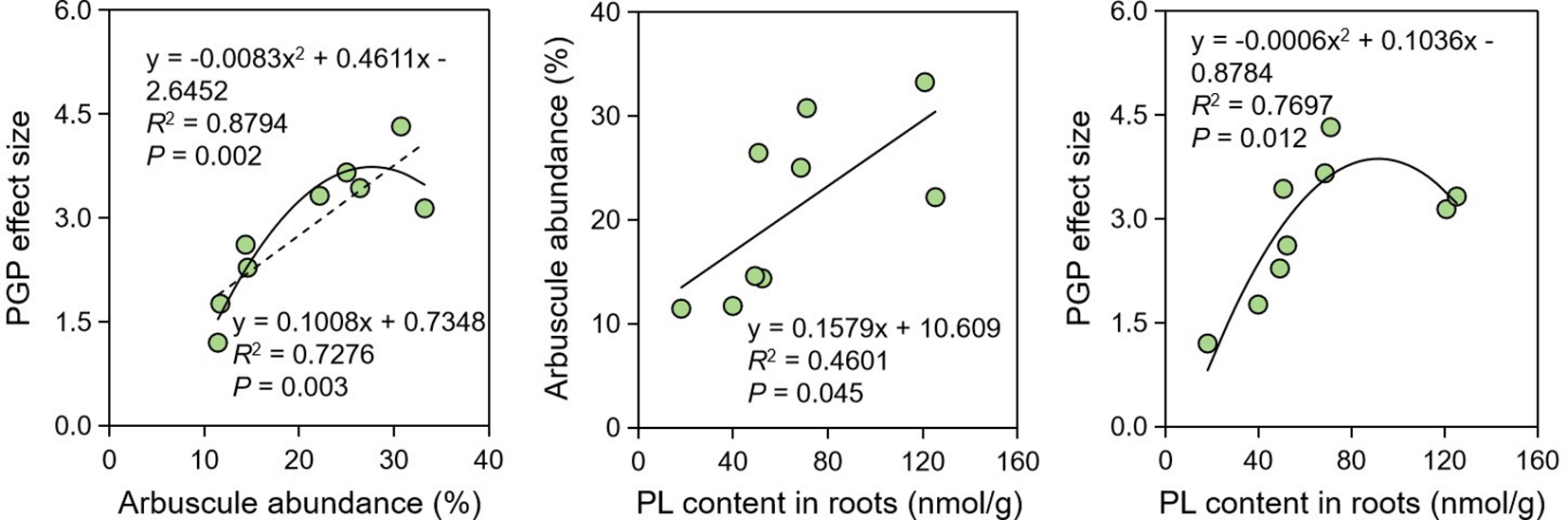
Regression analysis showing the relationships among PGP effect, arbuscule abundance and PL contents. PGP, plant growth promotion; PL, phospholipid.

Considering the influence of AM-specific lipids on arbuscule development and thus on PGP effects, we further explored the tripartite relationship among AM-specific PL contents, arbuscule abundance, and PGP effect size. Regression analysis indicates that their tripartite relationship were significant (*P* < 0.05) (**Figure 3**). The relationship between PGP effect size and arbuscule abundance was the strongest (*R* ^2^ = 0.8794, *P* = 0.002), while the relationship between arbusule abundance and AM-specific PL content was the weakest (*R* ^2^ = 0.4601, *P* = 0.045), which probably suggests that there were some factors besides PLs affecting the arbuscule formation.

**Figure 4:**
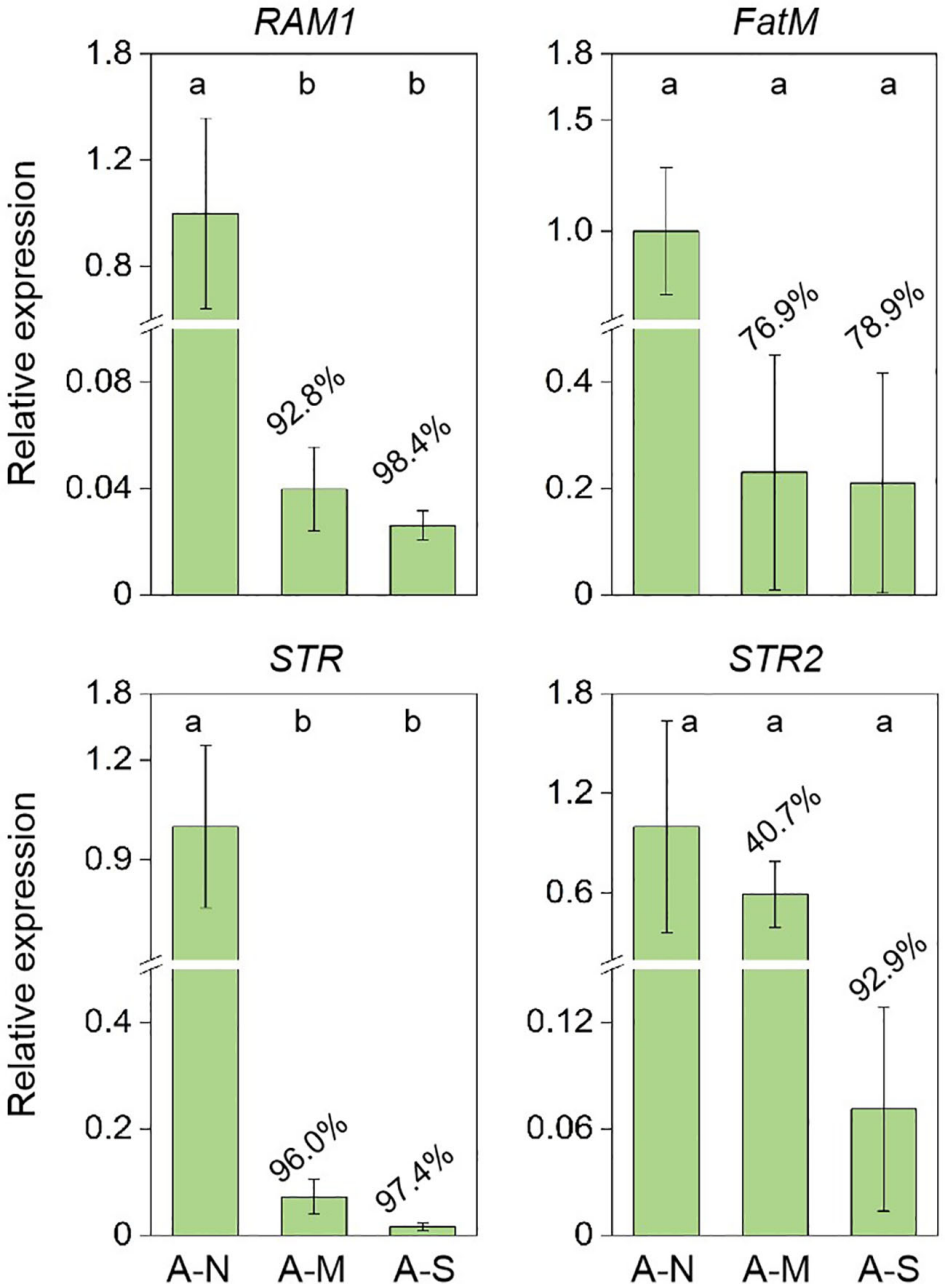
The inhibition of gene expression encoding for AM-specific lipid biosynthesis and delivery by drought stress. A-N, A-M, and A-S indicate the treatments of no drought stress, medium drought stress, and severe drought stress with AM inoculation, respectively. Different letters in each subplot indicate the significance by multiple range test (Tukey’s). The percentages indicate the decrease in the respective parameters of A-M or A-S compared to A-N.

The expression of genes encoding for the biosynthesis and delivery of AM-specific lipids were further investigated. Results showed that drought stress significantly inhibited the expression of *RAM1* (transcript factor of AM-specific lipid biosynthesis) and *STR* (transporter of AM-specific lipids) (**Figure 4**). The expression of *FatM* (AM-specific lipid biosynthesis) and *STR2* (transporter of AM-specific lipids) were also inhibited by 76.9%-78.9% and 40.7%-92.9%, respectively, due to drought stress (**Figure 4**).

### 3.4 Effects of AM fungal inoculation and different levels of drought stress on the lipid profiles in the roots

**Figure 5:**
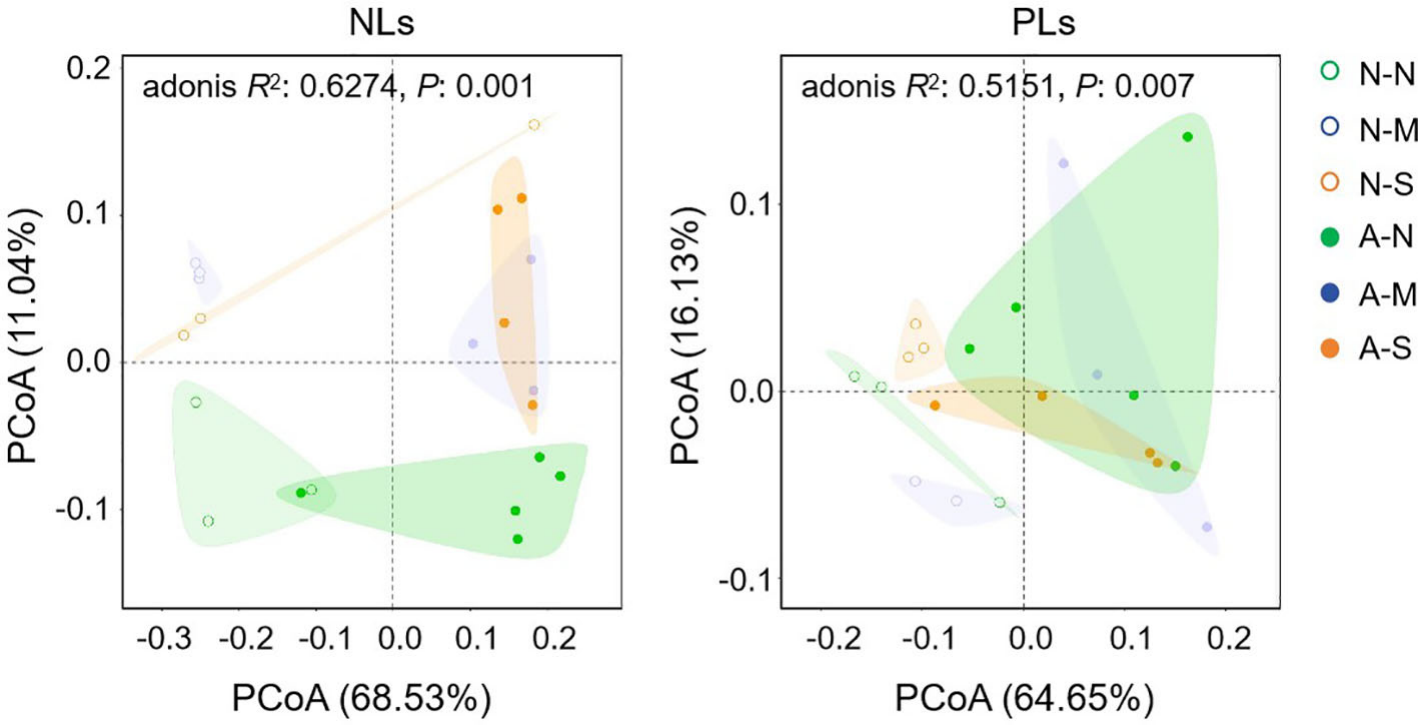
The effect of AM fungal inoculation and drought stress on the lipid profiles of *Poncirus trifoliata* roots as revealed by PCoA. N-N, N-M, and N-S indicate the treatments of no drought stress, medium drought stress, and severe drought stress with no AM inoculation, respectively, while A-N, A-M, and A-S indicate the treatments of no drought stress, medium drought stress, and severe drought stress with AM inoculation, respectively. NLs, neutral lipids; PLs, phospholipids.

To explore the involvement of non-AM-specific lipids in drought tolerance, we further investigated the effects of AM fungal inoculation and drought stress on lipid profiles in roots (**Supplementary Table S2**). PCoA indicates that both AM fungal inoculation and drought stress shifted the lipid profiles. Axis 1 and axis 2 totally explained 79.57% and 80.78% of the variations in lipid profiles among six treatments for NLs and PLs, respectively (**Figure 5**). Both NL profiles and PL profiles of non-AM treatments and AM treatments clearly separated from each other along axis 1. For NL profiles, treatments with no stress (N-N and A-N) separated well from those treatments with drought stress (N-M, N-S, A-M and A-S), but it was not the case for PL profiles (**Figure 5**). This result probably suggests that both AM fungal inoculation and drought stress regulated lipid metabolism, however, NLs and PLs showed different responsive patterns.

**Figure 6:**
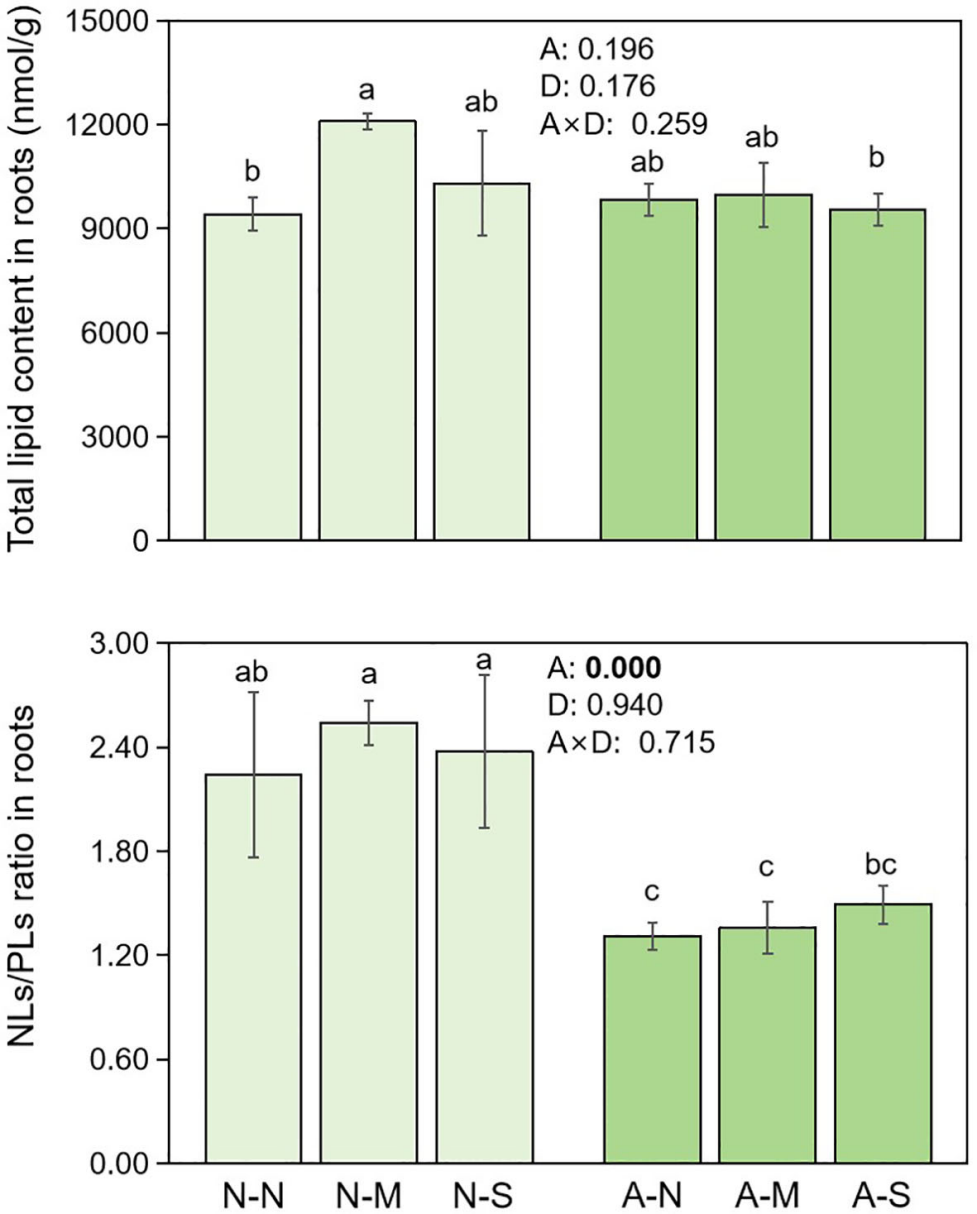
The effect of AM fungal inoculation and drought stress on the lipid contents and allocation in *Poncirus trifoliata* roots. N-N, N-M, and N-S indicate the treatments of no drought stress, medium drought stress, and severe drought stress with no AM inoculation, respectively, while A-N, A-M, and A-S indicate the treatments of no drought stress, medium drought stress, and severe drought stress with AM inoculation, respectively. Different letters in each subplot indicate the significance by multiple range test (Tukey’s). Values indicate the results of two-way ANOVA.

The total lipid contents (NLs and PLs) in roots were not affected by either AM fungal inoculation or drought stress (**Figure 6**). However, when lipid components (e.g., NLs vs PLs, saturated vs unsaturated) were considered, they were significantly affected by AM fungal inoculation and drought stress. Firstly, AM fungal inoculation significantly decreased NLs/PLs ratio in roots (**Figure 6**), indicating the decreased NL content or increased PL contents by AM fungal inoculation. More specifically, AM fungal inoculation significantly decreased the saturated NL contents, but significantly increased the saturated PL contents. Accordingly, AM fungal inoculation significantly increased the unsaturation rate of NLs. In contrast, drought stress significantly increased the unsaturated NL contents, and thus significantly increased the unsaturation rate of NLs (**Table 2**).

**Table 2:** The effect of AM fungal inoculation and drought stress on lipid contents in the roots of *Poncirus trifoliata*.

Mycorrhizal inoculation	Drought stress	NL-FAs (nmol/g DW)			PL-FAs (nmol/g DW)		
		Saturated	Unsaturated	Unsaturation rate (%)	Saturated	Unsaturated	Unsaturation rate (%)
N	N	4932.6 ± 594.8 ab	1485.2 ± 222.9 b	23.0 ± 0.7 c	2194.4 ± 246.2 c	811.5 ± 94.4 a	27.0 ± 0.8 a
	M	6296.7 ± 21.6 a	2373.5 ± 27.4 a	27.4 ± 0.2 bc	2518.5 ± 163.1 abc	914.0 ± 28.9 a	26.7 ± 0.7 a
	S	5083.0 ± 1795.6 ab	2188.6 ± 120.8 a	32.5 ± 6.0 ab	2326.8 ± 46.5 bc	711.4 ± 27.0 a	23.4 ± 0.7 a
A	N	3870.6 ± 334.9 b	1965.9 ± 95.8 a	34.0 ± 2.1 ab	3159.0 ± 270.1 ab	853.7 ± 145.5 a	21.3 ± 3.3 a
	M	3680.2 ± 215.7 b	2004.3 ± 115.5 a	35.3 ± 0.6 ab	3348.8 ± 331.7 a	955.2 ± 298.1 a	21.1 ± 4.1 a
	S	3261.3 ± 187.1 b	2201.6 ± 220.8 a	40.2 ± 3.3 a	3032.1 ± 309.0 abc	1066.3 ± 97.1 a	26.1 ± 0.7 a
Two-way ANOVA (*P* values)							
AMF (A)		**0.001**	0.750	**0.002**	**0.002**	0.251	0.188
Drought (D)		0.367	**0.011**	**0.046**	0.590	0.795	0.940
A × D		0.403	**0.049**	0.806	0.889	0.495	0.203

N and A in mycorrhizal inoculation column indicate non-inoculation and inoculation, respectively; N, M and S in drought stress column indicate no, medium and severe drought stress, respectively. NL, neutral lipid; PL, phospholipid; FAs, fatty acids. Unsaturation rate was calculated as the proportion of unsaturated FA content in the total FA content. Different letters in each column indicate the significance by multiple range test (Tukey’s).

The bold values mean significant differences.

Intriguingly, **Figure 6** indicates that AM fungal inoculation significantly reduced the NLs/PLs ratio, while drought stress exerted no influence. This result suggests that AM fungal inoculation promoted the allocation of lipids to PLs, namely, more lipids were used as biomembrane instead of as storage substance.

## 4 Discussion

Drought is the most common abiotic stress resulting in considerable loss in agricultural production, especially in the era of global climate change. Although AM fungi are reliable biotechnology to alleviate this stress, our study indicates that drought stress reduced the AM fungal functionality. The PGP effect size was 3.80 with no stress, significantly higher than those (2.82 and 2.06) with medium and severe drought stress. The significant relationship between PGP effect size and arbuscule abundance (**Figure 3**) indicates that the inhibited PGP effect size might be attributed to the reduced arbuscule abundance, which is the core structure of AM symbiosis enabling the nutrition exchange between plants and AM fungi (Luginbuehl and Oldroyd, 2017). The reduced arbuscule abundance by abiotic stresses has been reported under the stressed conditions of drought, low pH, salinity, heavy metals (Feng et al., 2020; Yang et al., 2020; Hu et al., 2021; Zhang et al., 2024). Specifically, Yin et al. (2024) revealed that drought stress not only retarded the arbuscule development but also accelerated the arbuscule senescence and degradation, thus leading to the decrease in arbuscule abundance in the roots of *P. trifoliata*. Since arbuscules are the key structure for P delivery from AM fungi to root cells in exchange for lipids and sugars (Rich et al., 2017), it is not surprising that the reduced arbuscule abundance by the medium or severe drought in this study eventually led to the decreased functionality of *R. irregularis* DAOM 197198. Therefore, it is concluded that drought stress can limit the AM fungal functionality because it may disrupt the nutrition exchange between symbionts by reducing the arbuscule abundance.

Since arbuscules are the key structure for PGP by AM fungi, we further explored the involvement of AM-specific lipids in the reduced arbuscule abundance by drought stress, because the arbuscule formation consumes a large amount of lipids to construct the huge surface areas (namely the bilayer membranes of PLs) (Dickson and Kolesik, 1999; van Aarle and Olsson, 2003). In this study, we found that the reduction in arbuscule abundance was in parallel with the decreased contents of AM-specific PLs in roots (**Figure 3**), clearly indicating the involvement of AM-specific lipids in the reduction. Both plant biomass and AM fungal biomass contain lipid components, however, the 16:1 ω5 component is exclusively contained in AM fungi, which is routinely used as the specific biomarker of AM fungi to estimate AM fungal biomass in diverse environments (Olsson and Lekberg, 2022). AM fungi do not possess the genes encoding the subunits of type I fatty acyl synthase required for the *de novo* synthesis of lipids, and thus exclusively depend on host plants for the access to these indispensable nutrition (Luginbuehl and Oldroyd, 2017; Jiang et al., 2017). In the long term of co-evolution, plants synthesize lipids specifically for their AM fungal symbionts via *FatM*, which is expressed only in colonized roots. Meanwhile, *RAM1* regulates the AM-specific expression of *FatM*, and *STR*/*STR2* specifically encode the delivery of lipids to AM fungi across the plant-fungal interface (Keymer and Gutjahr, 2018). In our study, the expression of all these four genes were greatly inhibited by drought stress, and accordingly, the AM-specific lipid contents in roots were also inhibited, which support the reduced the arbuscule abundance by drought stress.

Interestingly, drought stress did not inhibit the AM-specific lipid contents in soils, where AM fungal biomass mainly include external hyphae and spores. This might suggest that AM fungus exported more lipids in intraradical structures outside to the extraradical structure in response to the drought stress. Previous studies indicate that abiotic stresses, such as drought and low pH, can accelerate the arbuscule degradation inside the cortex cells (Feng et al., 2020; Yin et al., 2024), and probably trigger the transport of lipids from intraradical structures to the extraradical structures, especially for the sporulation (Feng et al., 2020). This might be one of the mechanisms by which AM fungi adapt to adverse conditions.

In this study, we found that not only the AM-specific lipids but also the non-AM-specific lipids responded to AM fungal inoculation and drought stress. This result suggests that non-AM-specific lipids in roots were closely associated with drought tolerance, with their causality to be explored. Wu et al. (2019) demonstrated that AM fungal inoculation significantly increased the unsaturation index of lipids, probably contributing to less oxidative damage by drought stress. Similarly, we found that AM fungal inoculation significantly increased the unsaturation ratio of NLs in this study. It is well-recognized that unsaturated PLs are essential to the fluidity and stability of plasma membrane (Harayama and Antonny, 2023). However, the function of unsaturated NLs, normally as storage lipids, remains elusive yet.

Since NLs and PLs are typically the storage lipids and membrane lipids, respectively, the allocation of lipids to NLs or PLs can be of some significance in response to abiotic stresses (Liu et al., 2019). In this study, more lipids in roots were allocated to PLs with AM fungal inoculation, probably implying the increased recovery or repair of damaged membranes due to AM fungal inoculation under drought stress. A great deal of studies showed that AM fungal inoculation promotes the antioxidase activity (e.g., SOD, POD, CAT) to protect the membranes from oxidative damage by oxygen radicals (Chandrasekaran and Paramasivan, 2022; Wang et al., 2024). Here, we propose an additional pathway by which AM fungal inoculation maintains the stability of membranes, namely the increased damage recovery of membranes due to prioritized allocation of lipids to PLs.

## 5 Conclusion

Lipid metabolism is pivotal to both AM colonization and drought tolerance in host plants. In this study, AM fungal inoculation increased the drought tolerance of *Poncirus trifoliata*, but the effect size decreased with drought severity. Regression analysis revealed that the effect size was closely related to arbuscule abundance. Lipidomic analysis indicated that drought stress inhibited the biosynthesis and delivery of AM-specific lipids to AM fungus, resulting in decreased arbuscule abundance, which was supported by the gene expression of *FatM*, *RAM1* and *STR/STR2*. Non-AM-specific lipids were also associated with drought tolerance, because AM fungal inoculation promoted the lipid allocation to PLs and increased the unsaturation rate of NLs. Taken together, these results demonstrate that lipid metabolism mediates the increased drought tolerance by AM fungal inoculation, and both AM-specific and non-AM-specific lipids are involved in this process with different mechanisms. These results point out that cultivars, whose AM-specific lipid biosynthesis and delivery processes are insensitive to drought stress, might benefit more from AM symbiosis under drought-stressed conditions. Therefore, it seems promising to incorporate *FatM*, *RAM1* and *STR/STR2* in the crop breeding program. On the other hand, the allocation of lipids to NLs or PLs and their inter-conversion in response to drought stress has yet to be fully elucidated, which merits further investigation.

## Data Availability

The original contributions presented in the study are included in the article/**Supplementary Material**. Further inquiries can be directed to the corresponding authors.
