# Supplementary material for: Drought stress reduces arbuscular mycorrhizal colonization of Poncirus trifoliata (L.) roots and plant growth promotion via lipid metabolism
Source: Front Plant Sci. 2024 Sep 20;15:1452202. doi: 10.3389/fpls.2024.1452202 (PMC11449747; doi:10.3389/fpls.2024.1452202)

**Figure S1. The plant growth as affected by AM fungal inoculation and drought stress.** AM: inoculation, CK: no inoculation; No drought stress: 18.0% soil water content, Medium drought stress: 13.5% soil water content, Severe drought stress: 9.0% soil water content.


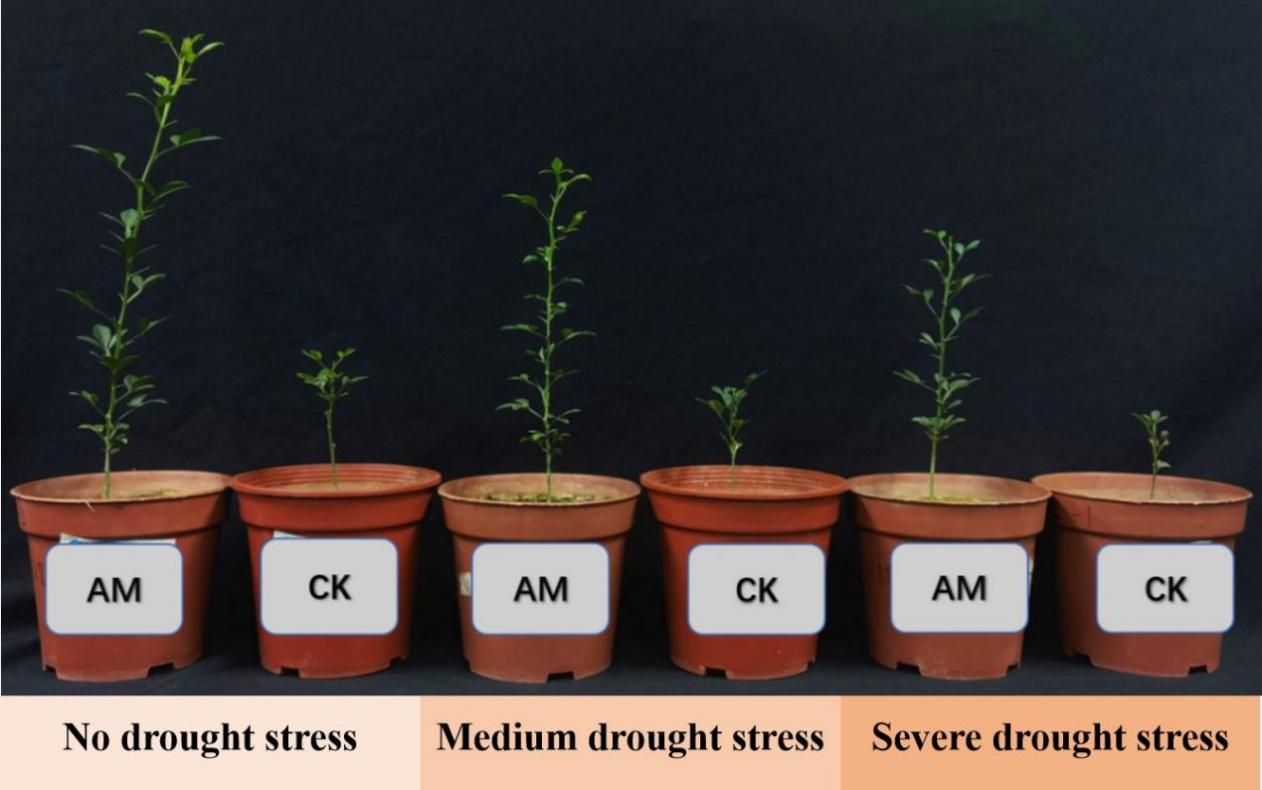

Supplement: Supplementary file 1 [file DataSheet1.docx]
