# Supplementary material for: Drought stress reduces arbuscular mycorrhizal colonization of Poncirus trifoliata (L.) roots and plant growth promotion via lipid metabolism
Source: Front Plant Sci. 2024 Sep 20;15:1452202. doi: 10.3389/fpls.2024.1452202 (PMC11449747; doi:10.3389/fpls.2024.1452202)
Supplement: Supplementary file 2 [file Table1.docx]

**Table S1. Sequences of the primers for the selected genes in this study.**

| Genes | Primer sequences (5'→3') | Amplicon size (bp) |
| --- | --- | --- |
| *actin* | F: CCGACCGTATGAGCAAGGAAA R: TTCCTGTGGACAATGGATGGA | 190 |
| *FatM* | F: CCGGTCTTCTCAGCAATGGT R: TTCAACAACCTCTCCCCAGA | 122 |
| *RAM2* | F: GCTGATTTGGTTCTGGGCAC R: ACCAAGTAACCCTCCTTGCAC | 145 |
| *STR* | F: ACTGGGTTTACGGAGTGCAA R: TCAAGGAACAACAGCGACGG | 76 |
| *STR2* | F: TCGCCGCTGATTTTCGTTTG R: TACGTGTTCCGAGTTGTCGTC | 103 |
